# The absence of a novel intron 19-retaining *ALK* transcript (*ALK*-I19) and *MYCN* amplification correlates with an excellent clinical outcome in neuroblastoma patients

**DOI:** 10.18632/oncotarget.24216

**Published:** 2018-01-12

**Authors:** Abdulraheem Alshareef, Meredith S. Irwin, Nidhi Gupta, Hai-Feng Zhang, Moinul Haque, Scott D. Findlay, Bo Kyung Alex Seong, Justine Lai, Mohammed Rayis, Sadeq Al-Dandan, Raymond Lai

**Affiliations:** ^1^ Department of Applied Medical Sciences, Taibah University, Medina, Saudi Arabia; ^2^ Department of Laboratory Medicine and Pathology, University of Alberta, Edmonton, Canada; ^3^ Division of Haematology-Oncology, Department of Pediatrics, The Hospital for Sick Children, University of Toronto, Ontario, Canada; ^4^ Department of Pathology and Laboratory Medicine, University of British Columbia, Vancouver, Canada; ^5^ Department of Oncology, University of Alberta, Edmonton, Canada; ^6^ Department of Anatomy and Cell Biology, Faculty of Medicine and Dentistry, University of Western Ontario, London, Canada; ^7^ Department of Medical Biophysics, University of Toronto, Toronto, Canada; ^8^ Department of Pediatric Oncology, King Fahad Medical City, Riyadh, Saudi Arabia; ^9^ Department of Anatomical Pathology, King Fahad Medical City, King Saud bin Abdulaziz University, Riyadh, Saudi Arabia; ^10^ DynaLIFE Medical Laboratories, Edmonton, Canada

**Keywords:** neuroblastoma, anaplastic lymphoma kinase, alk-expressing human cancers, intron-retention transcript, prognostic markers

## Abstract

ALK missense mutations are detected in 8% of neuroblastoma (NB) tumors at diagnosis and confer gain-of-function oncogenic effects. The mechanisms by which the expression of wild-type or mutant *ALK*, which is detectable in the majority of cases, is regulated are not well understood. We have identified a novel *ALK* transcript characterized by the retention of intron 19 (*ALK-I19*). *ALK-I19* was detected in 4/4 NB cell lines, but not other non-NB cells with *ALK* aberrations. The functional significance of *ALK-I19* was determined by specific siRNA knockdown of this transcript, which resulted in substantially decreased expression of the fully-spliced *ALK* transcripts (FS-*ALK*) and a significant reduction in cell growth. We also demonstrate that *ALK-I19* is a precursor of FS-*ALK*. *ALK-I19* was detected in 14/37 (38%) tumors from patients with newly diagnosed NB. *ALK-I19* expression correlated with undifferentiated histology and strong *ALK* protein expression detectable by immunohistochemistry. Importantly, patients with tumors that did not express *ALK-I19* and lacked MYCN amplification had an excellent clinical outcome, with 19/19 patients survived at 5-years. In conclusion, *ALK-I19* is a novel *ALK* transcript that likely represents a marker of undifferentiated NB cells. The absence of *ALK-I19* and *MYCN* amplification is a useful prognostic marker for NB patients.

## INTRODUCTION

*Anaplastic lymphoma kinase (ALK)*, which encodes a tyrosine kinase member of the insulin receptor superfamily, was initially identified as a potent oncogenic driver in anaplastic large-cell lymphoma (ALCL) [1, 2]. In ALCL, the tyrosine kinase of ALK is constitutively active, and this is directly resulted from the reciprocal chromosomal translocations fusing the portion of *ALK* that encodes the tyrosine kinase domain and different gene partners, with the *nucleophosmin* (*NPM*) gene being the most frequently implicated [2]. Consequently, multiple cellular signaling pathways and biochemical processes are deregulated, providing the molecular basis underlying the oncogenic effects of ALK [2]. Other than ALCL, *ALK* genetic alterations were identified in a variety of tumors including neuroblastoma (NB), myofibroblastoma and non-small cell lung cancer (NSCLC) [3]. These genetic alterations include chromosomal translocations involving fusion partners other than *NPM*, gene amplification and activating missense mutations, all of which are believed to result in constitutive activation of the ALK tyrosine kinase and the downstream cellular signaling pathways and biological processes [3]. In keeping with the pathogenetic importance of ALK in these human cancers, targeted inhibition of ALK using various tyrosine kinase inhibitors have shown therapeutic efficacy against subsets of ALK-positive tumors [3].

NB is the most common pediatric extra-cranial solid tumor and the survival for metastatic NB remains < 50% despite intensive multi-modality therapies [4]. Known prognostic factors at diagnosis that are associated with poor outcome include age of >18 months, *MYCN* amplification, advanced stage, undifferentiated histology and diploid DNA status [5]. ALK protein detectable by immunohistochemistry (IHC) has been reported in the majority of NB tumors [6–8], and it is believed that ALK carries pathogenetic importance in these cells [9–12]. *ALK* missense mutations are detected in 8% of NB tumors at diagnosis [13], but the prevalence appears to increase at recurrence [14–16]. The three hotspot mutations at residues 1174, 1245 and 1275 within the tyrosine kinase domain account for 85% of *ALK* missense mutations [13]. There is increasing evidence that these *ALK* mutations, with the *ALK*^F1174L^ mutation being the best characterized, are activating and oncogenic *in vitro* and *in vivo* [17]. The prognostic significance of *ALK* missense mutations and amplification varies in different studies; one analysis found a significant correlation between the presence of *ALK*^F1174L^ and *MYCN* amplification [7, 12, 15]. Nonetheless, the significance of ALK expression in the absence of *ALK* amplification and/or mutations is not well understood. Furthermore, a previous study reported that ALK protein expression is detectable in NB tumors regardless of the status of *MYCN* amplification [6]. Thus, the role and mechanisms of ALK expression in NB need to be further characterized.

In order to achieve a better understanding of the mechanisms that regulate the expression of *ALK* in NB, we examined the mRNA and protein expression in a cohort of NB cell lines and tumors. In particular, we assessed if *ALK* mRNA transcripts in NB cells retain evidence of intron 19 (I19), since ALK transcripts retaining portions of intron 19 were detected in subsets of melanoma and other tumors [18–23]. Our studies had revealed a unique *ALK* mRNA species in which the entire I19 was included in the mRNA transcripts. The biological and clinical significance of this *ALK* variant, labeled *ALK-I19*, are discussed.

## RESULTS

### Expression of *ALK-I19* in NB cell lines

We were particularly interested in the intron 19 region (I19) of *ALK*, since portions of I19 have been found in the mRNA transcripts of two *ALK* fusion genes, *EML4-ALK* and *PPFIBP1-ALK*, detectable in lung cancer and myofibroblastoma, respectively [18–22]. Of note, these I19-containing *ALK* transcripts have not been fully characterized and their significance is largely unknown. In a subset of melanoma, portions of I19 also was also detected in the *ALK* transcripts; importantly, these I19-containing *ALK* transcripts were found to be responsible for the aberrant expression of ALK in these tumors, as the I19 portions contain the transcription initiation sites [23]. To detect I19 in NB, we employed RT-PCR and an array of primer sets specifically designed to detect I19 (Table 1). Four NB cell lines, including NB1 (with amplified *ALK*), IMR32 (wild-type *ALK*), GOTO (wild-type *ALK*) and SK-N-SH (mutated *ALK*^F1174L^) were included in this study.

**Table 1:** Primers used in this study

Primer set #	Primer location	Expected product (bp)	Figures	Product	Method
1	Intron 19 to Exon 29	2884	1A, 2B	*ALK-I19*	RT-PCR
2	Exon 1 to Intron 19	3436	1A	*ALK-I19*	RT-PCR
3	Exon 1 to Exon 29	4286	1A, 2B	*FS-ALK*	RT-PCR
4	mid-point of Intron 19	99	1B, 3A	*ALK-I19*	qRT-PCR
5	Exon 24 to Exon 25	79	1B	Total *ALK*	qRT-PCR
6	Exon 18 to Intron 19	172	1C, 3D, 4B	*ALK-I19*	RT-PCR
7	Exon 18 to Exon 20	289	1C, 3D, 3E, 4D	*FS-ALK*	RT-PCR
8	Exon 20 to Exon 29	1636	Supplementary Figure 2A	Total *ALK*	RT-PCR
9	Exon 19 to Exon 20	236	4B	*FS-ALK*	RT-PCR

**Figure 1:**
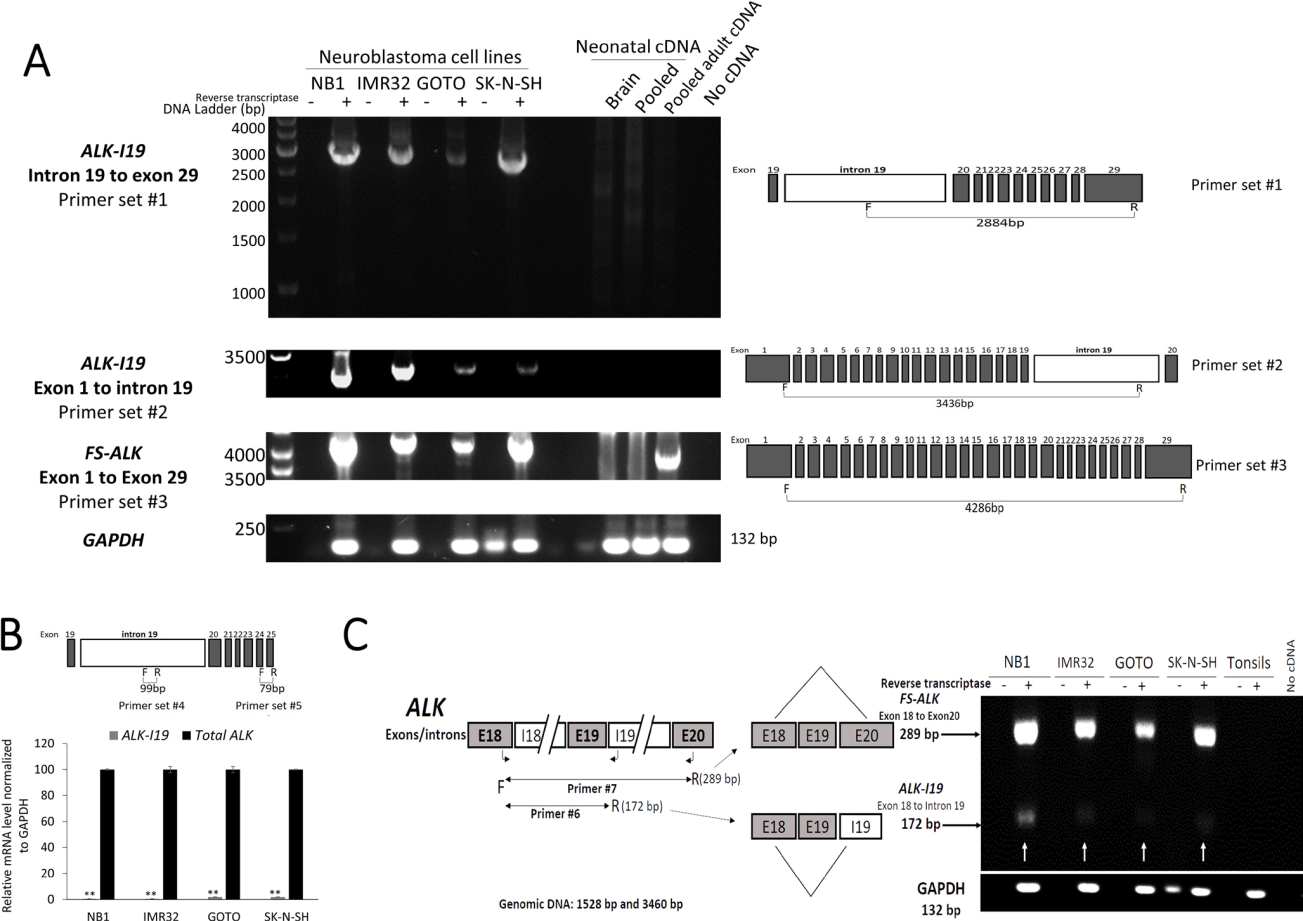
Expression of *ALK-I19* in NB cells (**A**) Detection of *ALK-I19* and *FS-ALK* transcripts in 4 NB cell lines (NB1, IMR32, GOTO and SK-N-SH) using RT-PCR and primer set #1-3. cDNA samples derived from neonatal brain, pooled normal adult tissues and pooled neonatal tissues were negative for *ALK-I19*. Triplicate experiments were performed and only one amplicon was identified in all runs. GAPDH served as the loading control. (**B**) *ALK-I19* was found to be expressed at a significantly lower level when compared to *FS-ALK*, as revealed by quantitative RT-PCR. Primer set #4 was designed to detect *ALK-I19* whereas primer set #5 was designed to detect all *ALK* transcripts. Data are presented as mean ± standard deviation and statistical analysis was performed using Student’s *t* test. The *p* value for all runs is < 0.01. (**C**) RT-PCR using both primer set #6 (to detect *ALK-I19*) and #7 (to detect *FS-ALK*) generated predominantly the *FS-ALK* amplicons, and this finding further supports the concept that *FS-ALK* is more abundant than *ALK-I19*. GAPDH was used as the loading control. Samples without added reverse transcriptase were used as the negative control.

Using primer set #1 (spanning from the 5′ end of I19 to the 3′ end of exon 29), we found an amplicon of ~2,800 bp in 4 of 4 cell lines (Figure 1A). The size of this amplicon matched that was expected for the combined length of all of the exons (i.e. exon 20 to exon 29) and the portion of I19 spanned by this primer set (i.e. 2,884 bp). Sequencing of this amplicon confirmed this impression (Supplementary Figure 1A). Triplicate experiments were run and the only one amplicon of ~2,800 bp was consistently found in all 3 runs.

To confirm the existence of the I19-containing *ALK* transcript (thereafter labeled as *ALK-I19*) in NB cells, we performed RT-PCR using another PCR primer set that spanned from exon 1 to the mid-region of I19 (#2; Table 1). As shown in Figure 1A, with the exception of NB1, we found only one detectable amplicon in all cell lines, and the size of ~3,500 bp matched that was expected for the combined length of all of the exons (i.e. exon 1 to exon 19) and the included portion of I19 spanned by the primer sets (i.e. 3,436 bp). Triplicate experiments were run and only one amplicon of ~3,500 bp was consistently detected. Sequencing of this amplicon confirmed this impression (Supplementary Figure 1B). Regarding NB1, the amplicons were at ~3,200 bp, since the *ALK* gene in these cells are known to have deletions of exon 2 and 3 [24]. Our sequencing studies confirmed this impression (not shown).

In order to substantiate the presence of *ALK-I19* transcript, we examined RNA-sequencing data of 7 NB cell lines from public available Cancer Cell Line Encyclopedia (CCLE) database. Six out of these 7 cell lines were known to express ALK mRNA/protein (i.e. IMR32, Kelly, NB1, SK-N-BE2, SK-N-DZ and SK-N-FI), while one cell line (i.e. SK-N-AS) was known to not express ALK mRNA/protein [25]. As expected, we found RNA-sequencing reads in the intron 19 of ALK in the 6 ALK-positive cell lines, however, no reads was observed in SK-N-AS (Supplementary Figure 1C). Moreover, we did not find RNA-sequencing reads in nearby introns (introns 18 and 20) (Supplementary Figure 1D), suggesting that RNA-sequencing reads from intron 19 are specific. Interestingly, although intron 19 shows RNA-sequencing reads, these reads were very low compared to only-exons reads. Specifically, quantification of intron 19 reads account for ~2% of ALK exons reads.

Using both primer set #1 and #2, we did not find evidence of *ALK-I19* in the cDNA samples derived from neonatal brain, pooled neonatal human tissues or pooled adult human tissues; in addition, no evidence of *ALK-I19* was found in a number of ALK-expressing, non-NB human cell lines including Karpas 299 and SupM2 (two ALK-positive anaplastic large cell lymphoma cell lines expressing NPM-ALK), H2228 (a lung cancer cell line expressing EML4-ALK) and U-87 MG (a glioblastoma cell line expressing wild-type full-length ALK)(Supplementary Figure 2A). Lastly, to confirm that the *ALK-I19* mRNA is indeed within NB cells, we performed RT-PCR using only 800 SK-N-SH cells laser micro-dissected out of paraffin-embedded tissue sections. As shown in Supplemental Figure 2B, *ALK-I19* was detectable.

### *ALK*-*I19* is present in a small amount compared to the fully spliced *ALK* (*FS-ALK*) transcripts

When we performed long-range RT-PCR using primer set #3 (from exon 1 to exon 29), we found only one band at ~4.3 kb, which is the approximate size of the *FS-ALK* transcript (i.e. 4,286 bp). Again, due to the deletions of exon 2 and 3, the amplicons from NB1 were slightly shorter than those from the other 3 cell lines. We speculated that the reason for not being able to detect *ALK-I19* with the long-range RT-PCR assay was because of its relatively low abundance, as compared to that of *FS-ALK*. In support of this concept, we performed quantitative RT-PCR (qRT-PCR) using primer set #4 (Table 1) covering a 99-bp segment in the mid-portion of I19 (Figure 1B), and we found that *ALK-I19* was present at a very low level, with the cycle number being 32, as compared to 26 for all *ALK* detectable with primer #5 spanning from exon 24 to exon 25 (Table 1). By performing qRT-PCR using serial dilutions of the same samples, we determined that the concentration difference between cycle 25 and cycle 32 is between 1:100 to 1:1000 (data not shown). A similar conclusion was obtained when we used primer set #6 and #7 (Table 1) simultaneously. Specifically, both primer sets shared the same 5′ primer, being located in exon 18. In set #6, the 3′ primer was located in I19, designed to detect *ALK-I19*. In set #7, the 3′ primer was located in exon 20, designed to detect *FS-ALK* transcripts. As shown in Figure 1C, the *FS-ALK* transcripts (~289 bp) were substantially more abundant than *ALK-I19* (~172 bp).

### *ALK*-*I19* is not derived from genomic DNA

We had considered the possibility that the *ALK-I19* was derived from genomic DNA, although several lines of evidence strongly argued against this possibility. First, as mentioned above, *ALK-I19* was detectable only in NB cell lines but not a number of other ALK-positive cell lines and normal tissues. As shown in Supplementary Figure 2A, a number of ALK-negative cancer cell lines as well as reactive tonsils were also negative with the same RT-PCR assay. Second, we performed DNA sequencing of the entire amplicon generated from primer set #1 and #2, and we did not identify introns other than that of I19 (data not shown). Third, we did not observe any bands in the negative control lanes (Figure 1), in which reverse transcriptase was omitted.

### siRNA knockdown of *ALK*-*I19* dramatically reduces the total *ALK* expression

**Figure 2:**
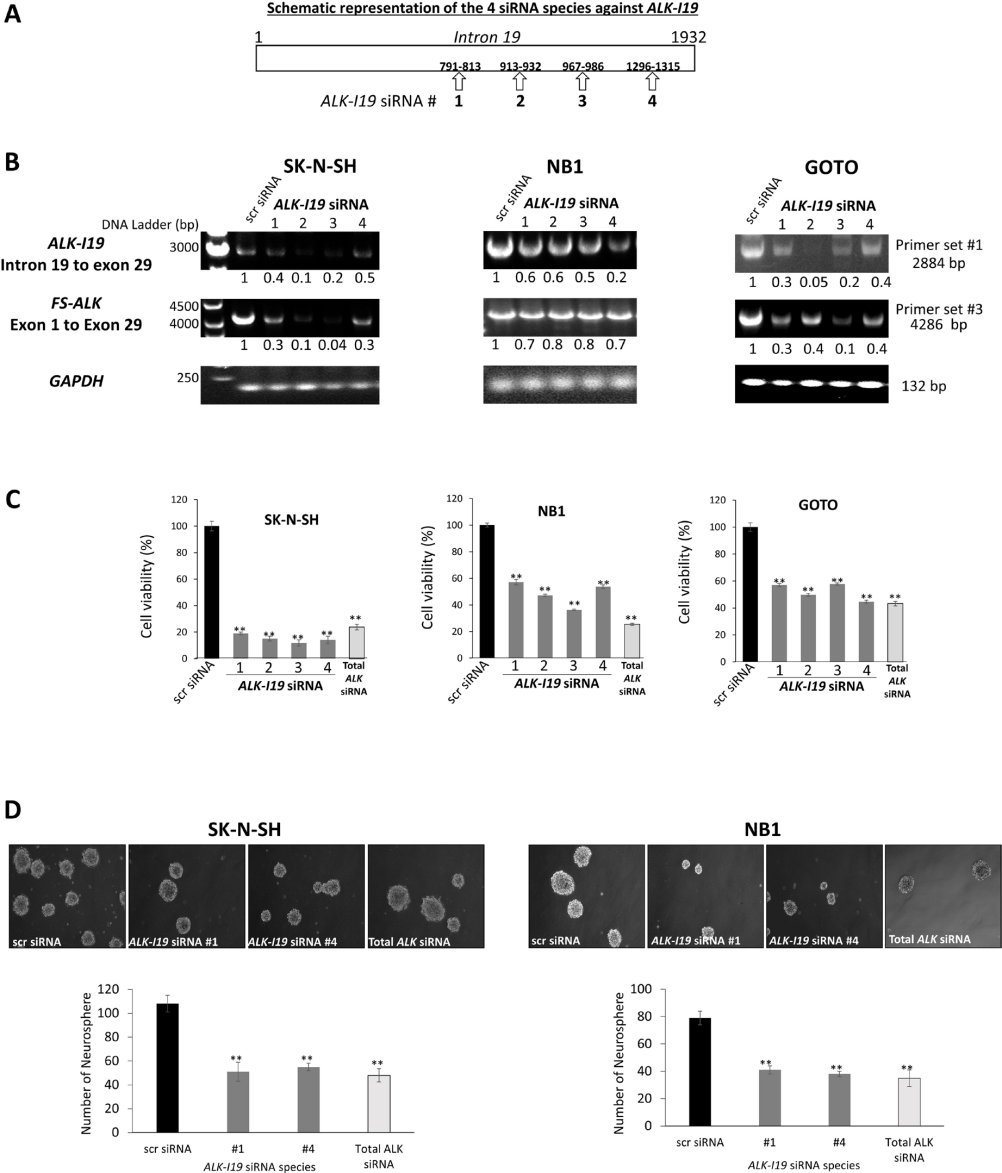
siRNA knockdown of *ALK-I19* decreases the expression of *FS-ALK*, cell growth and neurosphere formation in NB cells (**A**) Schematic representation of the four *ALK-I19* siRNAs used. (**B**) The expression levels of *ALK-I19* (primer set #1) and *FS-ALK* (primer set #3) were reduced 48 hours following siRNA knockdown of *ALK-I19* in three NB cell lines. GAPDH was used as the loading control for the RT-PCR assay. The densitometry value of each band was normalized to that observed with scrambled (scr) siRNA. (**C**) siRNA knockdown of *ALK-I19* significantly decreased cell growth at 72 hours, as assessed by MTS assay. Data is presented as mean ± standard deviation and statistical significance was determined using Student’s *t* test. The *p* value for all runs is < 0.01. Triplicate experiments were performed. (**D**) siRNA knockdown of *ALK-I19* significantly decreased neurosphere formation, measured at 2 weeks after the siRNA knockdown. Data is presented as mean ± standard deviation and statistical significance was determined using Student’s *t* test. The *p* value for all runs is < 0.01. Triplicate experiments were performed.

To assess the functional significance of *ALK-I19* in NB cells, we designed four intron 19-specific siRNA species, as illustrated in Figure 2A. The impact of these siRNAs on the expression levels of *ALK-I19* and *FS-ALK* was evaluated by using RT-PCR and two different primer sets, namely set #1 (to detect *ALK-I19*, ~2800 bp) and set #3 (to detect *FS-ALK*, ~4200 bp). As shown in Figure 2B, all 4 siRNA species were efficient in downregulating the expression level of *ALK-I19* in all 3 cell lines examined. Interestingly, all 4 siRNA species to knockdown *ALK-I19* were also efficient in downregulating the *FS-ALK*. This result was further confirmed using qRT-PCR (Supplementary Figure 3A). Western blot studies also confirmed the dramatic reduction in ALK protein expression as a result of *ALK-I19* knockdown (Supplementary Figure 3B).

### siRNA knockdown of *ALK*-*I19* decreases cell growth and soft agar colony formation

We then assessed whether the siRNA knockdown of *ALK-I19* affects the growth of NB cells. As shown in Figure 2C, the growth of 3 NB cell lines, as determined by MTS assay for 48 hours, was significantly decreased when *ALK-I19* was knocked down by using siRNA (*p* < 0.01), with the most dramatic effect observed in SK-N-SH cells. Interestingly, a similar growth inhibitory effect was observed when we used ALK siRNA targeting total ALK. In contrast, in Karpas 299, SupM2 and H2228, all of which have no *ALK-I19*, siRNA knockdown of *ALK-I19* had no appreciable effect on the expression of *FS-ALK*, ALK protein expression (data not shown) and cell growth (Supplementary Figure 3C). Similarly, siRNA knockdown of *ALK-I19* had no appreciable effect on the cell growth of two NB cell lines (i.e. SH-EP and SK-N-AS) that were known to not express ALK mRNA/protein [25] (Supplementary Figure 3C). Next, we examined the effect of siRNA knockdown of *ALK-I19* on tumorigenicity. As shown in Figure 2D, siRNA knockdown of *ALK-I19* significantly downregulated the neurosphere formation ability (i.e. > 50% reduction). Again, a similar neurosphere reduction was observed when we used ALK siRNA targeting total ALK. These results support the biological importance of *ALK-I19* on regulating the total expression of ALK and thus, its oncogenic effects on cell growth and tumorigenicity.

### *ALK-I19* is the precursor of the *FS*-*ALK* transcript

**Figure 3:**
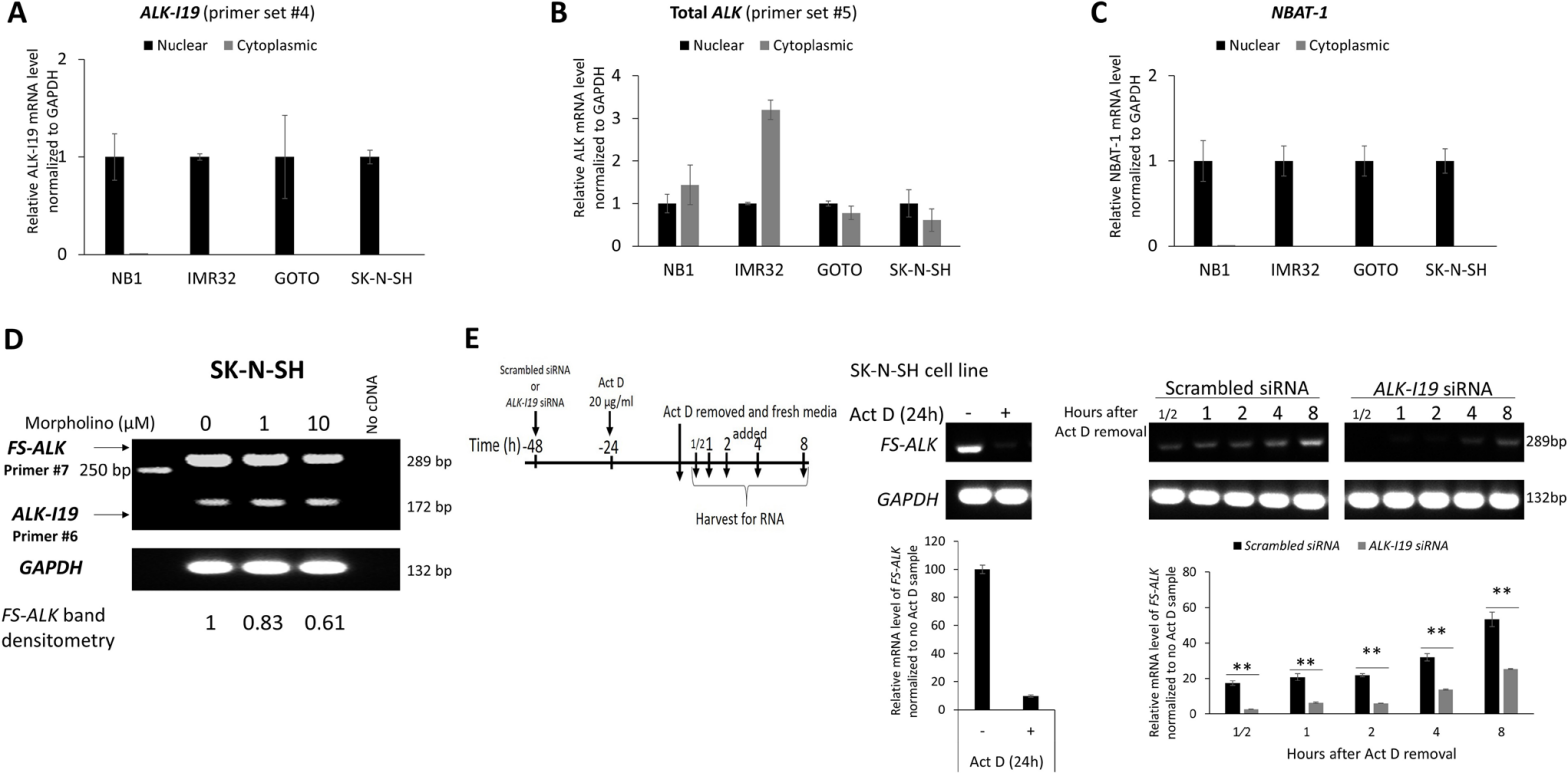
*ALK-I19* is the precursor of *FS-ALK* (**A**–**C**) Measurement of *ALK-I19* in the nuclear and cytoplasmic fractions of NB cells showed that *ALK-I19* is expressed only the nucleus. In contrast, total *ALK* was found in both the nuclear and cytoplasmic fractions. *NBAT-1* was used as the control for the nuclear/cytoplasmic fractionation efficiency [47]. (**D**) Inhibition of the splicing between exon 19 and intron of *ALK* 19 using morpholino appreciably decreased the level of *FS-ALK* at 48 hours. The densitometry value of *FS-ALK* bands was normalized to the cell sample not treated with morpholino. The level of *ALK-I19* was not dramatically changed. (**E**) SK-N-SH cells were first treated with either scrambled siRNA or *ALK-I19* siRNA for 24 hours. Subsequently, these cells were treated with 20 μg/ml Actinomycin D (Act D) for another 24 hours. After washing, cells were cultured in fresh growth media and RNA was harvested at different time points. This experiment was performed in the presence of either scrambled siRNA or *ALK-I19* siRNA. Cells treated with scrambled siRNA followed by actinomycin D expressed a substantial higher level of *FS-ALK* than cells treated with *ALK-I19* siRNA followed by actinomycin D. The densitometry value of each band was normalized to the cell sample receiving scrambled siRNA and no actinomycin D. Data is presented as mean ± standard deviation and the statistical significance was assessed using Student’s *t* test.

Based on our finding that siRNA knockdown of *ALK-I19* can efficiently down-regulate ALK expression, we hypothesized that *ALK-I19* is the precursor of *FS-ALK*. In support of this concept, we had collected additional evidence. First, we found that *ALK-I19* was confined to the nucleus in all 4 cell lines examined (Figure 3A); in contrast, *FS-ALK* was localized in both the nucleus and cytoplasm (Figure 3B). These results strongly suggest that *ALK-I19* is a non-coding transcript. *NBAT-1* (NB Associated Transcript 1), a long non-coding RNA that is exclusively localized in the nucleus (22), was used as a control for the efficiency of nuclear/cytoplasmic fractionation (Figure 3C). Second, when NB cells were treated with morpholino (an antisense oligomer) [26] that had been engineered to specifically block the splicing between exon 19 and intron 19, we found that the expression level of *FS-ALK* appreciably decreased at 48 hours, whereas the *ALK-I19* level remained largely unchanged (Figure 3D). Third, as shown in Figure 3E, cells treated with scrambled siRNA followed by actinomycin D for 24 hours expressed a substantial higher level of *FS-ALK* than cells treated with *ALK-I19* siRNA followed by actinomycin D.

### Expression of the *ALK*-intron 19 variant in NB tumors

To prove that the expression of *ALK-I19* is not restricted to cell lines, we surveyed the expression of this variant in 52 formalin-fixed/paraffin-embedded NB tumors derived from 37 patients, for whom the demographic data is summarized in Table 2. To facilitate the detection of *ALK-I19* in paraffin-embedded tissues, we employed primer set #6 (exon 18 to intron 19, Table 1) with which a relatively short amplicon will be generated (i.e. expected size, 172 bp). Similarly, to detect *FS-ALK* in these tissues, we employed a new primer set (#9) with which a relatively short amplicon (i.e. expected size, 236 bp, exon 19 to exon 20, Table 1) can be generated. Of note, RT-PCR using primer set #9 was not expected to detect *ALK-I19*, since the expected product (i.e. > 2 Kb) should not have survived the formalin-fixation.

**Table 2:** Clinicopathological characteristics of 37 neuroblastoma patients

Clinical parameters	Number of patients (*n* = 37)	Positive *ALK-I19* cases (*n* = 14)	*P* value	Positive *FS-ALK* cases (*n* = 15)	*P* value	Positive *ALK* by *IHC* (*n* = 14)	*P* value
**Gender**							
Male	24	8	*P* = 0.49	7	*P* = 0.08	8	*P* = 0.49
Female	13	6		8	6		
**Age at diagnosis**							
≤ 18 months	20	5	*P* = 0.10	5	*P* = 0.05	6	*P* = 0.33
> 18 months	17	9		10		8	
**COG-risk groups**							
Low-risk	9	0	*P* = 0.002^*^	1	*P* = 0.05	1	*P* = 0.016^*^
Intermediate-risk	11	3		4		3	
High-risk	17	11		10		10	
**INSS clinical stage**							
I	3	0	*P* = 0.04^*^	0	*P* = 0.32	1	*P* = 0.04^*^
II	1	0		0		0	
IVS	4	1		1		0	
III	11	3		5		3	
IV	18	10		9		10	
**Primary tumor site**							
Adrenal	23	11	*P* = 0.17	10	*P* = 0.74	11	*P* = 0.17
Extra-adrenal	14	3		5		3	
**Tumor Histology**							
≥ 50% areas with undifferentiated histology	21	13	*P* < 0.001^*^	10	*P* = 0.74	13	*P* < 0.001^*^
< 50% areas with undifferentiated histology	9	1		2		1	
GNB	5	0		2		0	
GN	2	0		1		0	
***MYCN***							
Amplified	10	6	*P* = 0.13	5	*P* = 0.71	6	*P* = 0.13
Not amplified	27	8		10		8	

Use Fisher's exact test for the P value (^*^ statistically significant at < 0.05). Abbreviations: Children's Oncology Group-risk groups, COG-risk groups; INSS, International NB Staging System; NB, neuroblastoma; GNB, ganglioneuroblastoma; GN, ganglioneuroma.

**Figure 4:**
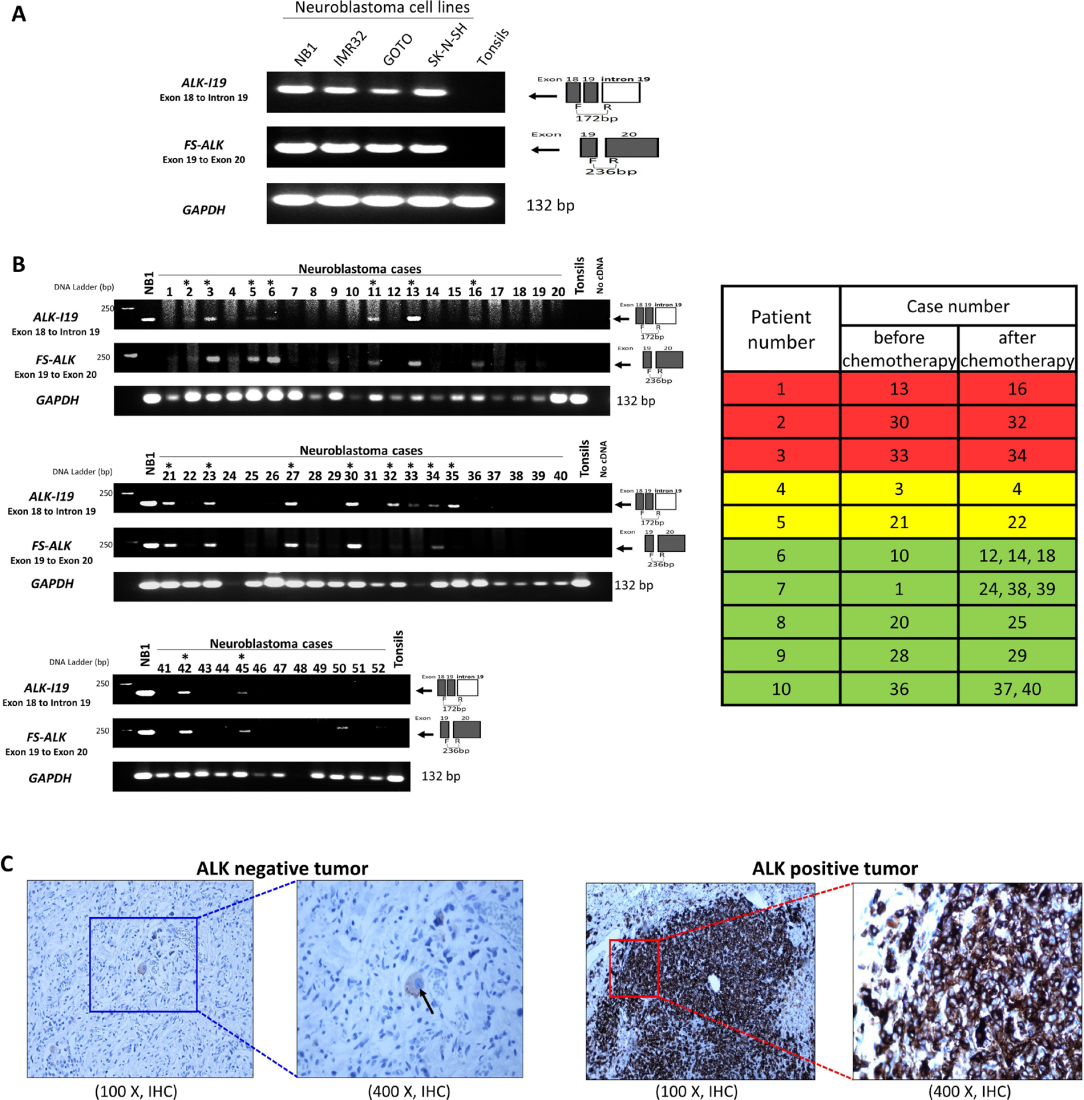
The expression of *ALK-I19* and FS-*ALK* in formalin-fixed/paraffin-embedded (FFPE) NB tumors (**A**) RT-PCR using primer set #6 (*ALK-I19*) and #9 (*FS-ALK*) was used to study 4 NB cell lines and a reactive tonsil. (**B**) RT-PCR using primer set #6 and #9 was used to detect the expression of *ALK-I19* and *FS-ALK* in 52 FFPE tumors derived from 37 patients. Asterisks indicate cases that were labeled *ALK-I19* positive, in which definitive *ALK-I19* bands could be identified. GAPDH was used as the loading control. The table on the right panel summarizes the 10 patients for whom pre- and post-chemotherapy tumor samples were obtained. Patient #1-5 had *ALK-I19* positive tumor at diagnosis. Patient #1-3 (highlighted in red) had *ALK-I19* positive tumors after chemotherapy whereas patient #4 and #5 (highlighted in yellow) had residual tumors that were *ALK-I19* negative. Patient #6-10 (highlighted in green) had *ALK-I19* negative tumors at diagnosis, and the residual tumors of all of these patients remained to be *ALK-I19* negative. (**C**) Immunohistochemical (IHC) detection of ALK showed heterogeneous staining intensity in NB tumors. On the left panel in which an ALK-negative tumor is illustrated, ALK immunostaining was found only in the ganglion-like cells (black arrow) and the intensity was weak. On the right panel in which an ALK-positive tumor is illustrated, intense ALK immunostaining was found in large tumorous areas of undifferentiated histology.

As illustrated Figure 4A, RT-PCR using primer #6 yielded the expected *ALK-I19* product in all 4 NB cell lines but not reactive tonsils. RT-PCR using primer set #9 yielded the expected *FS-ALK* in all 4 NB cell lines. As illustrated in Figure 4B, 14 of 37 (38%) tumor samples obtained at diagnosis showed definitive evidence of *ALK-I19*. With the exception of two cases (#33 and #35), these 14 positive cases also showed a readily detectable *FS-ALK* band. Of the remaining 23 tumors, *ALK-I19* was undetectable, and *FS-ALK* was not detectable in 20 cases, with tumor #9, #28 and #50 showing faint bands. The association between *ALK-I19* and *FS-ALK* is highly statistically significant (*p* = 0.0001).

We then correlated these RT-PCR findings with IHC results, which are illustrated in Figure 4C. Thirteen out of the 14 tumors that expressed *ALK-I19* also showed strong ALK expression, as defined by > 50% of tumor containing areas showing strong ALK immunostaining, a criterion used in a previous publication [27]. Of the 23 tumors that were negative for *ALK-I19*, only one case was ALK-positive by IHC. Overall, we have identified a high statistical correlation between the presence of *ALK-I19* detectable by RT-PCR and ALK protein expression detectable by IHC (*p* < 0.0001). Importantly, we observed that strong ALK staining intensity was largely restricted to the areas of undifferentiated histology, a finding that is also shared by another group [28]. Accordingly, we found a significant correlation between the expression of *ALK-I19* and tumors with > 50% undifferentiated histology (*p* = 0.01). Of note, ganglion-like cells in the well-differentiated tumorous areas consistently expressed ALK weakly (Figure 4C). Thus, only the intensely ALK-positive areas, typically found only in the areas of undifferentiated histology, were included for IHC scoring.

To determine whether the expression of *ALK-I19* is associated with other known features of NB, we correlated the expression of *ALK-I19* with a number of clinical and biological prognostic factors, as summarized in Table 2. Although *ALK-I19* did not significantly correlate with age, *ALK-I19* was more likely to be present in the older patients (i.e. >18 months, 64% versus 36%, *p* = 0.10). *ALK-I19* was significantly associated with stage 4 diseases (*p* = 0.04). Importantly, *ALK-I19* was significantly associated with the high-risk group based on Children's Oncology Group-risk classification (*p* = 0.002) [29]. *ALK-I19* was more common in cases with *MYCN* amplification but the correlation was not statistically significant (6/10 versus 8/27 cases, *p* = 0.13).

**Figure 5:**
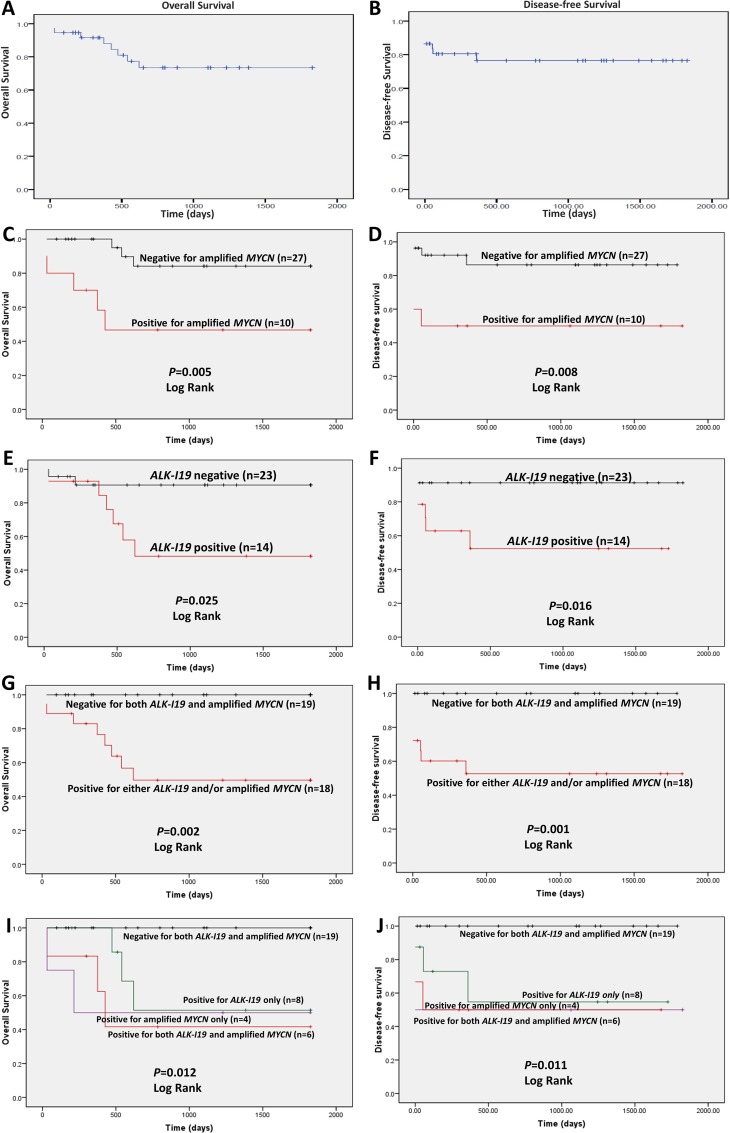
The prognostic significance of *ALK-I19* and MYCN amplification in 37 NB patients based on the overall survival (left panel) and the disease free survival (right panel) Kaplan–Meier survival is illustrated for the following: (**A** and **B**) the entire cohort; (**C** and **D**) patients with *MYCN* amplification versus those without; (**E** and **F**) patients with *ALK-I19* versus those without; (**G** and **H**) patients with *ALK-I19* and/or *MYCN* amplification versus those negative for both genetic markers; (**I** and **J**) patients with *ALK-I19* versus patients with *MYCN* amplification versus patients with both genetic markers versus those negative for both genetic markers.

In order to determine whether *ALK-I19* expression correlates with clinical outcome, we determined the overall survival (OS) and disease-free survival (DFS) data for our cohort (*n* = 37). The median overall survival for this cohort was 33.1 months (range, 0 to 96 months, median follow-up 63 months). The 5 years OS and DFS for our cohort is shown in Figure 5A and 5B. As expected, patients with *MYCN* amplification (*n* = 10) survived significantly shorter compared to those with normal *MYCN* (*n* = 27) (*p* = 0.005) (Figure 5C and 5D). Patients with *ALK-I19*–positive tumors (*n* = 14) had inferior OS and DFS compared to those with *ALK-I19*–negative tumors (*p* = 0.025 and 0.016, respectively) (Figure 5E and 5F). Importantly, when we compared the clinical outcome of those with neither of these two abnormalities and those with both/either of these two abnormalities, we found a high statistical significance (*p* = 0.002 for OS and *p* = 0.001 for DFS) (Figure 5G and 5H). Similar significance was observed when we performed this analysis for *FS-ALK* expression (Supplementary Figure 4A–4D). Importantly, at the end of 5 years, all 19 patients with tumors carrying neither of these two markers survived, in contrast with only 10 of 18 patients with tumors carrying one or both of these two markers. Moreover, when we compared the clinical outcome of patients with *ALK-I19* and *MYCN* amplification, either one of these two aberrations, or neither, we found statistical significance indicating the poor outcome associated with both *ALK-I19* and *MYCN* amplification (*p* = 0.012 for OS and *p* = 0.011 for DFS) (Figure 5I and 5J). Similar significance was observed when we performed this analysis using ALK expression detectable by IHC (Supplementary Figure 4E-4H). In summary, the absence of both *ALK-I19* and *MYCN* amplification strongly correlated with an excellent clinical outcome in our cohort. Importantly, the inclusion of *ALK-I19* allowed the identification of a subset of NB patients with inferior outcome, despite the lack of *MYCN* amplification. In other words, *ALK-I19* appears to be a useful prognostic value, especially used in combination with *MYCN* amplification.

Lastly, to determine whether *ALK-I19* expression changed following treatment, we identified 10 of 37 patients within our cohort who had sequential tumor samples after chemotherapy. Five of these 10 patients were positive for *ALK-I19*. Of these 5 patients, 3 remained to have *ALK-I19*–positive in the post-chemotherapy residual tumors. All of these 3 tumors were confirmed to be ALK-positive by IHC (i.e. > 50% of the tumor), and the ALK-positive areas were largely restricted to the undifferentiated areas. For the remaining two patients whose tumors were *ALK-I19*–positive at diagnosis, the residual tumors became *ALK-I19* negative, and ALK-negative by IHC and interestingly, most of the cells in these two post-chemotherapy tumor samples showed significant differentiation. The median overall survival was shorter for the patients whose tumors were *ALK-I19*–positive pre- and post-chemotherapy as compared to those whose tumors changed from positive to negative following chemotherapy (510 versus 1294 days). Of the five patients with *ALK-I19*–negative tumors at diagnosis, the post-chemotherapy residual tumors remained negative for *ALK-I19* expression and ALK expression by IHC. The median survival of the five *ALK-I19*–negative patients was 800 days. Of note, three out of the five *ALK-I19*–negative patients carry *MYCN* amplification.

## DISCUSSION

The key finding of this study is the identification of a novel *ALK* transcript, *ALK-I19*, which was detectable in 4/4 NB cell lines and in 38% of our cohort of NB tumors at time of diagnosis. *ALK-I19* is the precursor of *FS-ALK*, and abrogation of this transcript dramatically down-regulated *FS-ALK* as well as ALK protein expression. In NB tumors, we found that the expression of *ALK-I19* significantly correlates with > 50% areas of undifferentiated histology, and a number of other known prognostic factors such as stage 4 disease, high-risk group and *MYCN* amplification. Importantly, all 19 patients whose tumors were negative for *ALK-I19* and/or *MYCN* amplification survived > 5-years after the initial diagnosis. Based on our cell line studies as well as RNA-sequencing data analysis, its appears that the expression of *ALK-I19* is not dependent on the mutational status of ALK, since it was identified in our 4 NB cell lines and the 6 analyzed cell lines that carry wild-type *ALK*, amplified *ALK* or mutated *ALK*.

To our knowledge, the inclusion of I19-containing *ALK* transcripts has been reported in 6 ALK-expressing tumors described in five studies published between 2008–2013 (17–21). Of these 6 cases, 5 were non-small cell lung cancer expressing *EML4-ALK* and one case was myofibroblastoma expressing *PPFIBP1-ALK*. Sequencing data is available for these 6 cases; all include a portion of the 3′ end of I19 ranging from 12 bp to 117 bp. In all 6 cases, the 5′ end of the included I19 directly connects with the fusion gene partners (i.e. *EML4* or *PPFIBP1*) and 3′ end of the included I19 connects with exon 20 of *ALK*. In 5 cases, RT-PCR studies revealed that these I19-containing *ALK* transcripts are the only *ALK* mRNA species detectable; in the remaining one case, both I19-containing and the typical *ALK* fusion transcripts without any introns were identified. In none of these studies, the ALK protein was fully analyzed and whether the I19 was represented at the protein level is unknown. The significance of the inclusion of I19 in these *ALK* transcripts is also largely unknown. In a more recent study of melanomas published in 2015, *Wiesner et al*. reported the existence of I19-containing *ALK* transcripts in which only the 3′ end of I19, approximately 400 bp in length, represents the beginning of the *ALK* transcript and producing a novel initiation transcription site; these aberrant *ALK* transcripts were found in 11% of their cohort of melanomas [23]. Importantly, these transcripts and the resulting alternative initiation site lead to the production of truncated ALK proteins (ALK ^ATI^) that are oncogenic (22). Interestingly, while portions of I19 appear to have the propensity of being included in the *ALK* transcripts expressed in some cancer cells, we are not aware of any example in which the inclusion of other *ALK* introns is described. In this regard, we speculate that the exclusive retention of the full I19 in this setting may be linked to the unique biochemical characteristics of this specific intron. In support of this concept, almost all *ALK* translocations in ALK-expressing human cancers have breakpoints located at the junction between intron 19 and exon 20 [30].

*ALK-I19* is unique in several aspects. In contrast with these I19-containing *ALK* transcripts described above, our identified *ALK-I19* contains the entire I19 that is ~2 kb. Secondly, in contrast with the I19-containing *ALK* transcripts reported previously, which appeared to be the only *ALK* transcript expressed in the cancer cells, *ALK-I19* was the minor *ALK* mRNA species, being over-shallowed by the abundant *FS-ALK* in NB cells. Third, *ALK-I19* is a precursor of *FS-ALK*, and we had no evidence that the intron 19 was translated into proteins detectable by Western blots using two different anti-ALK antibodies. In contrast, in the previous reports of the I19-containing transcripts, it is highly likely the portions of I19 were translated, since these I19-containing transcripts were the only *ALK* transcripts detectable. Lastly, the two I19-containing ALK transcripts were found in the two fusion ALK genes (namely *EML4-ALK* and *PPFIBP1-ALK*); in contrast, *ALK-I19* was found to be expressed in NB cells expressing *ALK* without fusion partners.

Our data suggests that *ALK-I19* is unique to NB cells. Specifically, it was not found in normal tissues nor other ALK-expressing human cancer cells. Furthermore, this aberrant transcript was not detectable in the RNA samples derived from U-87 MG cells, a glioblastoma cell line that was derived from malignant astrocytes [31]. Taken together, it is possible that *ALK-I19* represents a relatively specific biological marker of undifferentiated NB cells. In other words, NB tumors expressing a relatively high proportion (i.e. 50%, as shown in our study) of areas of undifferentiated histology will express *ALK-I19* detectable by using our RT-PCR assay, whereas tumors in which the areas of undifferentiated histology represent only a minor component will have no detectable *ALK-I19*. It is of interest to test this hypothesis by including more NB tumors and other forms of ALK-expressing human cancer cells in the future studies.

The retention of specific introns or portions of introns has been described in normal cells in plants and animals [32]. The expression of intronic mRNA species is particularly high in the brain, especially the fetal brain [33]. The biological significance of these unusual transcripts has been examined, but it is not completely understood. Nonetheless, in a number of scenarios, intron-retained transcripts serve as reservoirs, which can boost protein production upon specific stimuli [34, 35]. For example, one study has identified an unusual intron 3-containing *ApoE* mRNA species that is detectable in cortical and hippocampal neuronal cells [31]. It was suggested that the expression of this specific transcript contributes to the relatively rapid upregulation of the ApoE protein in response to cellular stress [31]. In other words, intron 3-retaining *ApoE* is believed to provide a ‘*ApoE* mRNA reserve pool’, which can be called upon to synthesize a large of ApoE protein within a relatively short timeframe. Whether the existence of intron-retaining mRNA species in cancer cells provides the same purpose requires further investigations.

Intron-retaining transcripts also have been described in cancer cells, although their significance has not been extensively studied [34]. In a review paper by *Wong et al.* [34], RNA sequencing has identified intron retention in thousands of genes expressed in tumors of lung, breast, kidney, prostate and head/neck; the biological functions of these transcripts are largely unknown. A few studies have provided specific examples. *RET*, which encodes another tyrosine kinase, can be expressed as an intron 2-retaining variant that is detectable in 19% of pheochromocytomas [36]. Intron 4-retaining *CCDN1* expressed in prostate and esophageal cancers was found to translate into a truncated cyclin D1 protein which has oncogenic effects [37, 38]. With respect to the role of entire I19 retention (*ALK-I19*), we speculate that the ‘*ApoE* model’ may be more applicable. In our model, *ALK-I19*, the precursor of *FS-ALK*, is stored in the nuclei of NB cells, possibly predominantly in those that are undifferentiated. In response to adverse events, such as hypoxia or exposure to chemotherapeutic agents, cell survival signals may be induced including the ability to upregulate the expression of ALK, a protein known to promote survival in cancer cells [3]. This may provide one of the biochemical explanations to the prognostic significance of *ALK-I19* in NB.

Our finding that ALK expression detectable by IHC correlates with prognosis is in keeping with the conclusions of 4 previously published studies [6, 7, 27, 28]. It is important to point out that, despite the fact that different anti-ALK antibodies were employed in each of these 4 published studies, and that the scoring criteria used in these studies were not uniform, the prognostic significance of ALK immunostaining scores was identified in these studies.

However, all four studies used a cut-off of 50% ALK-positive immunostained tumor cells, which formed the basis of the scoring criteria used in our current study. In our experience, the staining intensity, in addition to the percentage of tumor cells showing ALK immunostaining, is important. In fact, 3 of these 4 published papers incorporated the staining intensity in their scoring. The rationale is related to the fact that the ganglion-like cells in the differentiated areas are consistently weakly ALK-positive. By including the most intensely stained areas only, one can essentially exclude the well-differentiated areas and take into the account the poorly differentiated areas exclusively, which are strongly ALK positive by IHC and likely express the *ALK-I19*. With these considerations, it is not surprising that we have identified a strong correlation between the expression of *ALK-I19* and the proportion of poorly differentiated areas, as well as the frequency of observing > 50% tumor areas showing strong ALK immunostaining.

One of the most important findings of this study is related to the potential clinical utility of *ALK-I19* expression in primary NB tumors. The expression of this aberrant *ALK* transcript, being found approximately one-third of the NB patients, significantly correlated with a shorter survival. Our analyses have also shown that *ALK-I19* may have additional prognostic power when used in combination with *MYCN* amplification. Specifically, in the absence of both *ALK-I19* and *MYCN* amplification, all 19 (100%) patients survived and 10 of these 19 (55%) patients were disease-free at 5 years. In comparison, only 10 of 18 patients with either *ALK-I19* or *MYCN* amplification survived at 5 years (56%) and only 4 of these 18 patients (22%) have no evidence of disease at 5 years. Perhaps the most interesting subset of patients are those with tumors that lacked *MYCN* amplification but were positive for *ALK-I19* expression (*n* = 8). The survival of this subset of patients was found to be comparable to those with tumors harboring *MYCN* amplification alone. Based on these findings, we conclude that the detection of *ALK-I19* can substantially improve the prognostic value of *MYCN*, and we propose that these findings to be further validated in a larger cohort. Although *ALK-I19* was found to correlate with ALK expression by IHC, we believe that detection of *ALK-I19* by RT-PCR is more advantageous than detection of ALK by IHC, since the detection of *ALK-I19* is less subjective and prone to the variability related to the use of the IHC methods and antibodies employed. Furthermore, this assay can be performed on routine-fixed/paraffin-embedded tissues, and this facilitates its adoption in routine pathology laboratories.

In conclusion, we have identified *ALK-I19*, a novel *ALK* transcript expressed exclusively in a subset of NB. *ALK-I19* is the precursor of *FS-ALK*, and our data suggests that it is likely a biological marker of undifferentiated NB cells. Furthermore, the prognostic significance in our small cohort suggests that the status of *ALK-I19* may be used together with *MYCN* amplification to further identify groups of patients with particularly poor outcome and may be useful in risk stratification. Further studies will be required to determine whether the *ALK-I19* expression has any impact on response to ALK inhibitors or other therapies.

## MATERIALS AND METHODS

### Clinical samples

Human NB samples were collected between September 2006 and May 2014 directly after surgical resection at the Department of Anatomical Pathology of King Fahad Medical City (KFMC), Saudi Arabia. The 37 “biopsy” specimens were pre-chemotherapy (30 NB, 5 GNB and 2 GN). Ten out of the 37 patients had additional samples/tumor resection specimens harvested post-chemotherapy. The cases were selected based on a clear pathological diagnosis, follow-up data, and had not received previous local or systemic treatment. The histological characterization was based on the International Neuroblastoma Pathology Classification (INPC) and the clinicopathological staging was performed in accordance with the International NB Staging System (INSS) [39]. Differentiation status was evaluated by two pathologists (S. A. and R. L.). Detailed clinical information of the NB patients is described in Table 2. Of note, patients were risk stratified and treated according to the Children's Oncology Group (COG)-risk groups [40]. The study was approved by the Institutional Review Board of the KFMC. Cytogenetic studies of the *MYCN* gene amplification status were performed by FISH as described previously [39].

### Cell lines and cDNAs

The four ALK-positive NB cell lines (NB1, IMR32, GOTO and SK-N-SH) used in this study were kind gifts from Dr. Roseline Godbout (Department of Oncology, University of Alberta, Canada). The non-small cell lung cancer cell line, H2228, was a kind gift from Dr. Ming Tsao (Ontario Cancer Institute). The characteristics of the ALK^+^ALCL cell lines (Karpas 299 and SupM2) have been previously described [41]. All cell lines were maintained in RPMI 1640 (Life Technologies, Grand Island, NY, USA) supplemented with 10% fetal bovine serum (FBS) (Life Technologies, Grand Island, NY, USA). All cell lines have been authenticated using short tandem repeat DNA profiling (from TCAG Genetic Analysis Facility, Toronto, CA). cDNA templates generated from normal human neonatal brain (catalog # cDNA-hsa-08), normal human fetal lung and spleen (catalog # cDNA-hsa-09), and normal human adult brain, lung and testis (catalog # cDNA-hsa-15) were purchased from Biosettia Inc. (Biosettia, San Diego, CA, USA). cDNA template generated from OVCAR3 cells was a gift from Dr. YangXin Fu (Department of Oncology, University of Alberta, Canada). cDNA template generated from BCPAP cells was a gift from Dr. Todd McMullen (Department of Oncology, University of Alberta, Canada).

### RNA-sequencing data analysis

The CCLE RNA-sequencing data (.bam files) for NB cell lines were downloaded from NCI GDC Data portal (https://gdc-portal.nci.nih.gov/) [42]. Integrative Genomics Viewer (IGV) was used to visualize RNA-sequencing data and to generate screenshots of RNA-sequencing reads at indicated regions. To quantify RNA-sequencing reads, all the alignments from selected regions (i.e. intron 19) were exported and counted. Through the CCLE Terms of Access, we declare that, “those who carried out the original analysis and collection of the data bear no responsibility for the further analysis or interpretation of it.”

### RNA isolation, reverse transcription PCR (RT-PCR), quantitative real-time PCR (qRT-PCR) and western blot analysis

Total RNA was extracted from cell lines with the RNeasy Mini Kit (Qiagen, Valencia, CA) and from NB tissue blocks with the PureLink FFPE RNA Isolation Kit (Invitrogen, Carlsbad, CA, USA). Trace DNA was removed by treatment with TURBO DNA-free Kit (Ambion, Life Technologies, Carlsbad, California, USA). Reverse Transcription (RT) reactions were performed with 0.5 to 2 μg of total RNA using Superscript First-Strand Synthesis System Kits (Invitrogen, Carlsbad, CA, USA). The RT-PCR parameters include an initial denaturing step at 95°C for 3 minutes followed by 35 cycles of denaturing at 95°C for 45 seconds, annealing at 56°C for 30 seconds and extension for 30 seconds to 5 minutes (1 kb/1 min), followed by a final extension step at 72°C for 10 minutes. PCR product was analyzed by agarose gel electrophoresis using a 1% (w/v) agarose gel prepared in 1X TAE buffer (40 mM Tris, 20 mM acetic acid, 1 mM EDTA) containing 1:10,000 SYBR Safe DNA gel stain (Invitrogen). The quantitative real-time PCR (qRT-PCR) include an initial denaturing step at 95°C for 10 minutes followed by 40 cycles of denaturing at 95°C for 15 seconds, annealing/extension at 60°C for 60 seconds. Expression of total *ALK*, *ALK-I19* or *NBAT-1* was normalized to *GAPDH* and relative expression determined using the ΔΔ-CT method [43].

Primers were designed using Primer3 software and were purchased from Invitrogen. The sequences of the primers used in this study include:

Primer set #1: (F- ACGTGCTCGGCAATTTACAC) & (R- GGGCCCAGGCTGGTTCATGC); Primer set #2: (F- AGAAGAAGGCGTCGGAAGTG) & (R- ATGTG CTCAGTTCCCTCCTC); Primer set #3: (F- AGAAGAA GGCGTCGGAAGTG) & (R- GGGCCCAGGCTGGTTC ATGC); Primer set #4: (F- ACGTGCTCGGCAATTTA CAC) & (R- ATGTGCTCAGTTCCCTCCTC); Primer set #5: (F- CCTGTGGCTGTCAGTATTTGG) & (R- GGA CAGGTCAAGAGGCAGTT); Primer set #6 & #7: (F- GTCTCCTGCATTGTGTCACC) & (R- ATTCA GTCCTGCCTTCCTGC) & (R- TTTTCCGCGGCACCT CCT); Primer set #8: (F- CCCTGAGTACAAGCTG AGCA) & (R- GGGCCCAGGCTGGTTCATGC); Primer set #9: (F- CCTCTCTGTGGTGACCTCTG) & (R- TTTT CCGCGGCACCTCCT); Primer set for *NBAT-1*: (F- G CTCTACATGACGGGAAAGC) & (R- AAGCAGCCT CTGATCCATGA); Primer set for *GAPDH*: (F- GGTCT CCTCTGACTTCAACAGCG) & (R- ACCACCCTGTT GCTGTAGCCAA).; *Glyceraldehyde-3-phosphate dehydrogenase* (*GAPDH*) mRNA was used as an internal control. Sequencing was performed at The Applied Genomics Centre (TAGC), University of Alberta. For western blot analysis, protein extraction and ALK antibody staining were performed as described previously [44]. Laser micro-dissection was performed using the PALM microbeam (Zeiss, Oberkochen, Germany).

### Immunohistochemistry (IHC)

The immunohistochemical studies were performed using the anti-ALK mouse antibody Ab-1 (Clone 5A4; NeoMarkers) on 4 μm thick paraffin sections at the Cross Cancer Institute, Edmonton, Alberta, Canada. The heat-induced epitope retrieval was performed by heating in Tris-EDTA buffer, pH 9.0. An ALK-translocated t(2;5) anaplastic large-cell lymphoma sample was used as a positive control. Reactive tonsils tissue was used as a negative control. ALK was considered positive when more than 50% of tumor cells were strongly reactive with anti-ALK, and this criterion is similar to that reported previously [6, 27].

### Nuclear/cytoplasmic RNA fractionation

Nuclear and cytoplasmic RNAs were isolated using the PARIS Kit (Ambion, Life Technologies, Carlsbad, California, USA) according to the manufacturer's instructions. RNA was quantified, and RT-PCR was performed using ABI (Invitrogen, Carlsbad, CA, USA) according to the manufacturer's instructions. Trace DNA was removed as described above. Reverse Transcription (RT) reactions were performed with 250 ng of total RNA using Superscript First-Strand Synthesis System Kits (Invitrogen, Carlsbad, CA, USA).

### Small interfering RNA (siRNA) transfection, reagents, cell growth analysis and neurosphere formation assay

Four small interfering RNAs (siRNA) species were designed against *ALK-I19*, specifically targeting intron 19, and were purchased from Sigma-Aldrich. The sequences for *ALK-I19* siRNAs are 1. 5′-AAUCUGAUCACGGUCGGUCCAUU-3′ 2. 5′-AAU UUUGUUCUGGCUUCCA-3′ 3. 5′-AGUGAUAAUGGU CACUCAC-3′ 4. 5′-AAGAUCCCAGCUGCACCCU-3′. siRNAs were transfected using Lipofectamine RNAiMAX (Invitrogen). Cell growth was performed as previously described [45, 46]. For the total ALK siRNA knockdown experiments, ALK specific ON-Target Plus SMARTpool small interfering RNA (siRNA) and scramble control were purchased from Thermo Scientific (Chicago, USA). Briefly, cell lines were transfected with either scrambled siRNA or *ALK-I19* or total *ALK* siRNA for 72 hours. The cell viability was then measured using the 3-(4,5-dimethylthiazol-2-yl)-5-(3-carboxymethoxyphenyl)-2-(4-sulfophenyl)-2H-tetrazolium, inner salt (MTS) assay (Promega, Madison, WI, USA) following the manufacturer's protocol. For neurosphere formationassay, NB1 and SK-N-SH cells were transfected with scrambled siRNA or *ALK-I19* or total *ALK* siRNA. After 24 hours, cells counted and 50,000 cells seeded into Neural Proliferation Medium (NeuroCult™ proliferation Kit, StemCell Technologies) containing recombinant hFGF (20 ng/ml, Peprotech) and recombinant hEGF (20 ng/ml, Peprotech). Cells were cultured for two weeks before the colonies were quantified.

### Antisense morpholino oligonucleotides

NB cells were transfected with morpholino antisense oligonucleotides using the Electro square electroporator BTX ECM 800 (225 V, 8.5 ms, 3 pulses) (Holliston, MA, USA) according to the manufacturer's instructions. Briefly, 5 million cells were resuspended into 500 μl of media with 1 or 10 μM of either *ALK* exon 19/intron 19 oligonucleotides (5′- GGCTCTGTGCACTCACCAATCATG -3′ or a standard control oligonucleotides and electroporated with the BTX ECM 800. Cells were harvested after 48 hours for RNA and protein extraction.

### Statistical analysis

The statistical analyses were performed using either the SPSS V.13.0 statistical software package or the Graphpad Prism6 to compute statistical significance in NB patient groups. Overall survival and disease-free survival curves were plotted using the Kaplan–Meier method and compared using the log-rank test. Student two-tailed test or two-tailed Fisher's exact test were used to generate significant differences between two group means and 2×2 correlations, respectively. *P* values were considered statistically significant at less than 0.05.

## SUPPLEMENTARY MATERIALS FIGURES


