# Supplementary material for: The absence of a novel intron 19-retaining ALK transcript (ALK-I19) and MYCN amplification correlates with an excellent clinical outcome in neuroblastoma patients
Source: Oncotarget. 2018 Jan 12;9(12):10698–713. doi: 10.18632/oncotarget.24216 (PMC5828214; doi:10.18632/oncotarget.24216)
Supplement: Supplementary file 1 [file oncotarget-09-10698-s001.pdf]

The absence of a novel intron 19-retaining *ALK* transcript (*ALK*-I19) and *MYCN* amplification correlates with an excellent clinical outcome in neuroblastoma patients

SUPPLEMENTARY MATERIALS

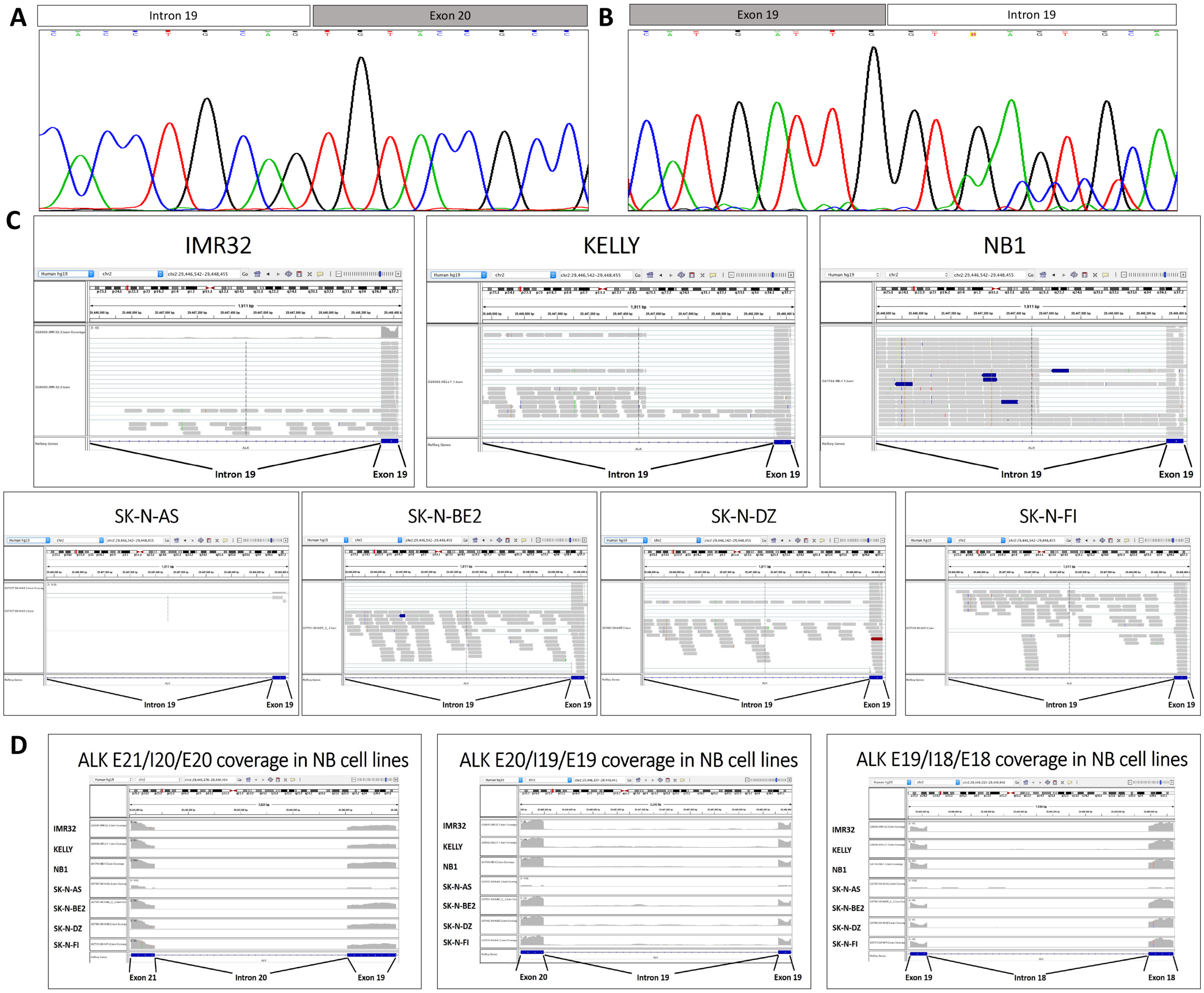

Supplementary Figure 1: Electropherograms for *ALK-I19* in the intron 19-exon 20 and the exon 19-intron 19 junction. (**A**) DNA sequencing of the intron 19-exon 20 junction present in the amplicons generated by RT-PCR and primer set #1. (**B**) DNA sequencing of the exon 19-intron 19 junction present in the amplicons generated by RT-PCR and primer set #2. The findings of both analyses support the concept that the entire I19 was included in the *ALK-I19* transcript. (**C**) RNA-sequencing reads detected in the intron 19 of ALK gene in 6 of 7 NB cell lines. (**D**) No RNA-sequencing reads detected in the two nearby introns, introns 18 and intron 20.

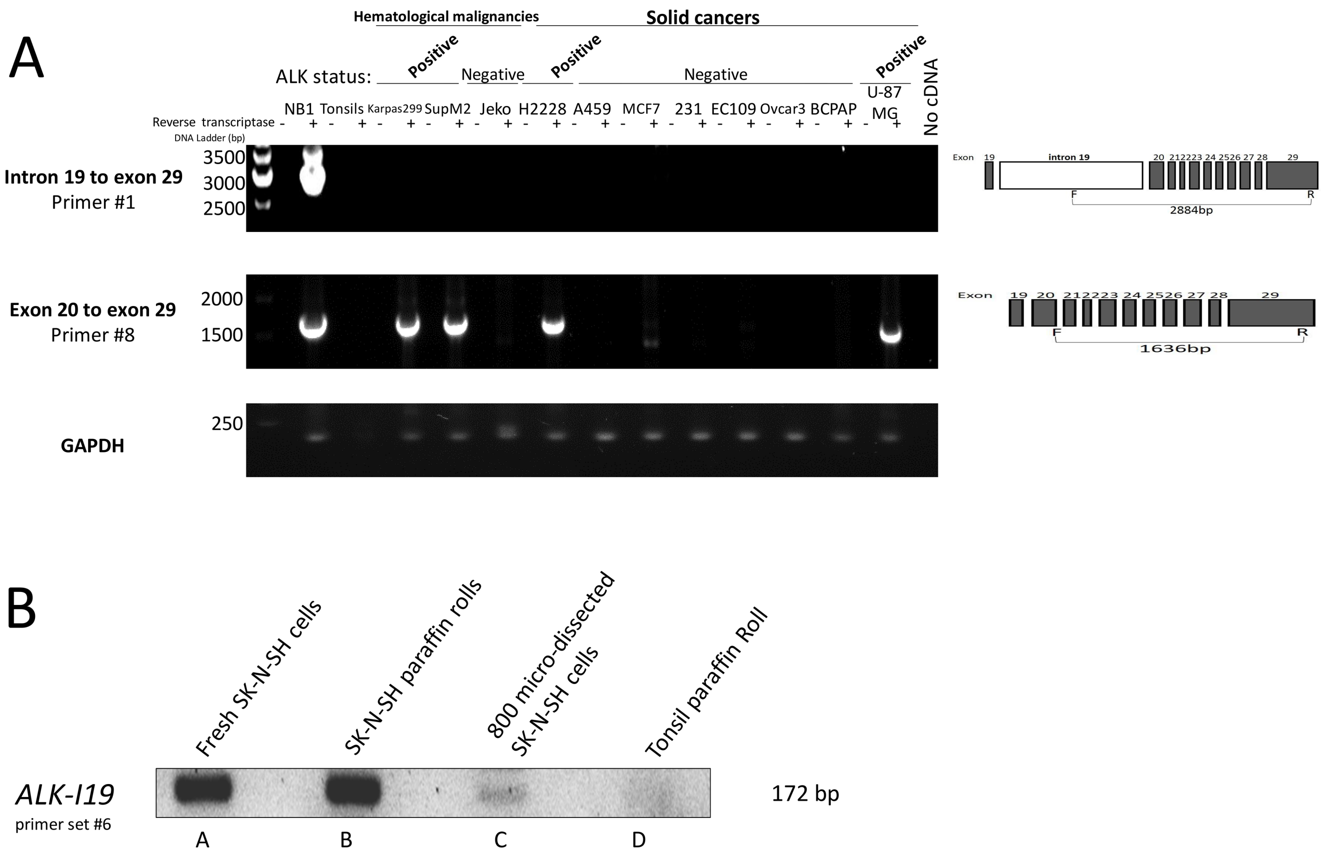

Supplementary Figure 2: ALK-I19 is not detectable in non-NB, ALK-expressing as well as ALK-negative human cell lines. (**A**) RT-PCR using primer set #1 was used to detect ALK-I19, and the result was positive for NB1 (positive control) and negative for reactive tonsils (negative control). Karpas 299 and SupM2 (ALK+ anaplastic large cell lymphoma cells expressing NPM-ALK), H2228 (a lung cancer cell line expressing EML4-ALK), U-87 MG (a glioblastoma cell line expressing wild-type full-length ALK), six ALK-negative cancer cell lines were all negative. The ALK-negative cell lines include Jeko (mantle cell lymphoma), A459 (lung adenocarcinoma), MCF7 (estrogen receptor-positive breast cancer), MB-MDA-231 (labeled as 231, triple-negative breast cancer), EC109 (esophageal squamous cell carcinoma), Ovcar3 (ovarian cancer) and BCPAP (thyroid cancer). (**B**) RT-PCR using primer set #6 was performed to detect *ALK-I19*. Lane A was derived from RNA extracted from fresh SK-N-SH cells, a neuroblastoma cell line carrying *ALK-I19*. Lane B was derived RNA extracted from curls of a paraffin-embedded, formalin-fixed SK-N-SH cell blocks. Lane C was derived from RNA extracted from 800 single, paraffin-embedded SK-N-SH cells that were laser micro-dissected using the PALM microbeam (Zeiss, Oberkochen, Germany). Lane D was derived from RNA extracted from curls of a paraffin-embedded, formalin fixed benign tonsil block. All RNA extraction was performed using the Recover™ total nucleic acid isolation kit from Invitrogen (Burlington, Ontario, Canada). Our results confirmed that the *ALK-I19* signals were from within the SK-N-SH cells.

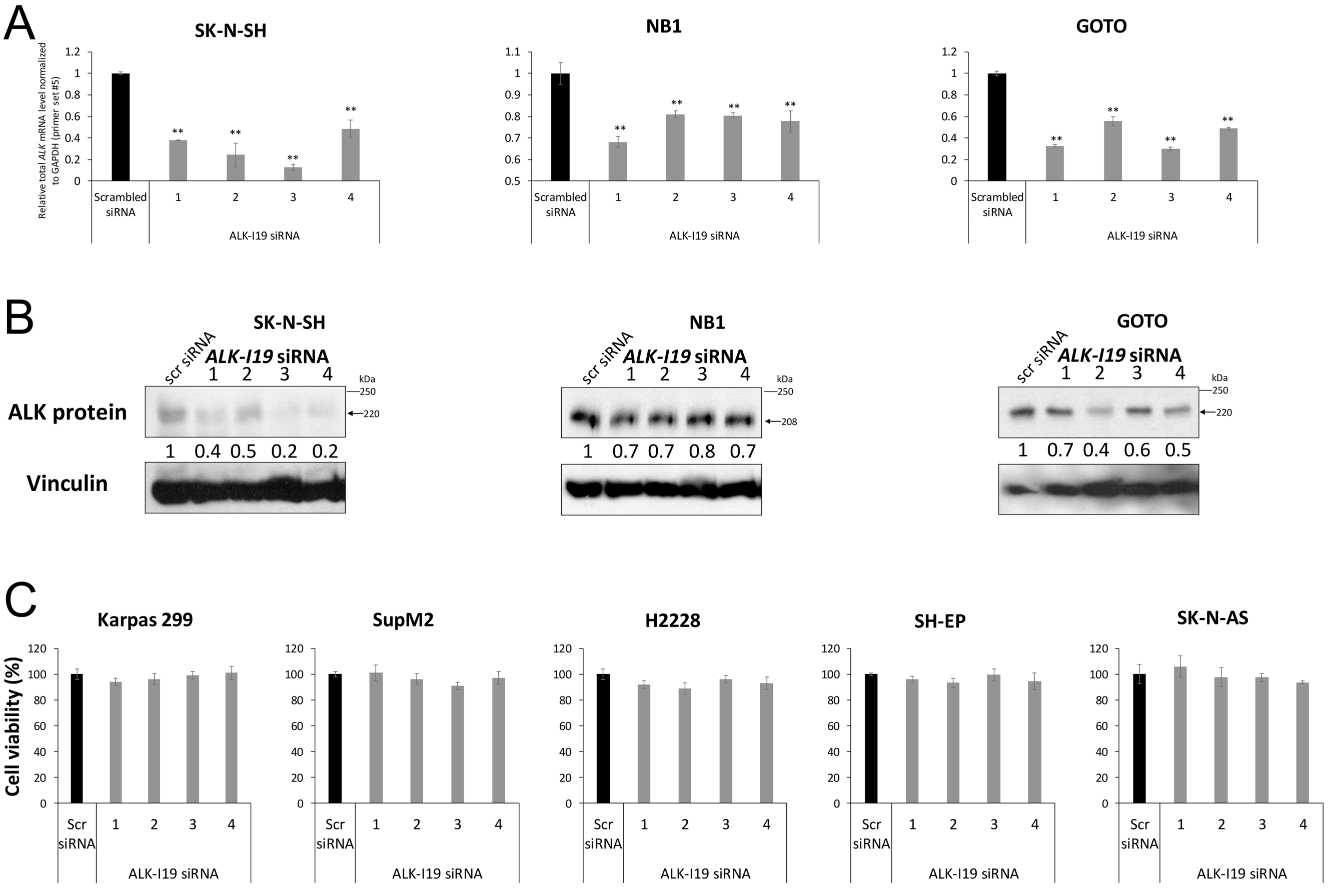

Supplementary Figure 3: siRNA knockdown of *ALK-I19* decreases the expression of *FS-ALK*, ALK protein and neither affect cell growth of ALK+ non-NB cells or ALK- NB cells. (**A**) Using quantitative RT-PCR (primer set #5), the expression level of all *ALK* transcripts was reduced 48 hours following siRNA knockdown of *ALK-I19* in three NB cell lines. Data are presented as mean ± standard deviation and statistical analysis was performed using Student's *t* test. The *p value* for all runs is < 0.01. (**B**) The expression level of ALK protein was reduced 48 hours following siRNA knockdown of *ALK-I19* in three NB cell lines. Vinculin was used as the loading control for the western blots. The densitometry value of each band was normalized to that observed with scrambled (scr) siRNA. (**C**) siRNA knockdown of *ALK-I19* did not significantly affect the cell growth of Karpas 299, SupM2, H2228, SH-EP and SK-N-AS. Cell growth was assessed using the MTS assay.

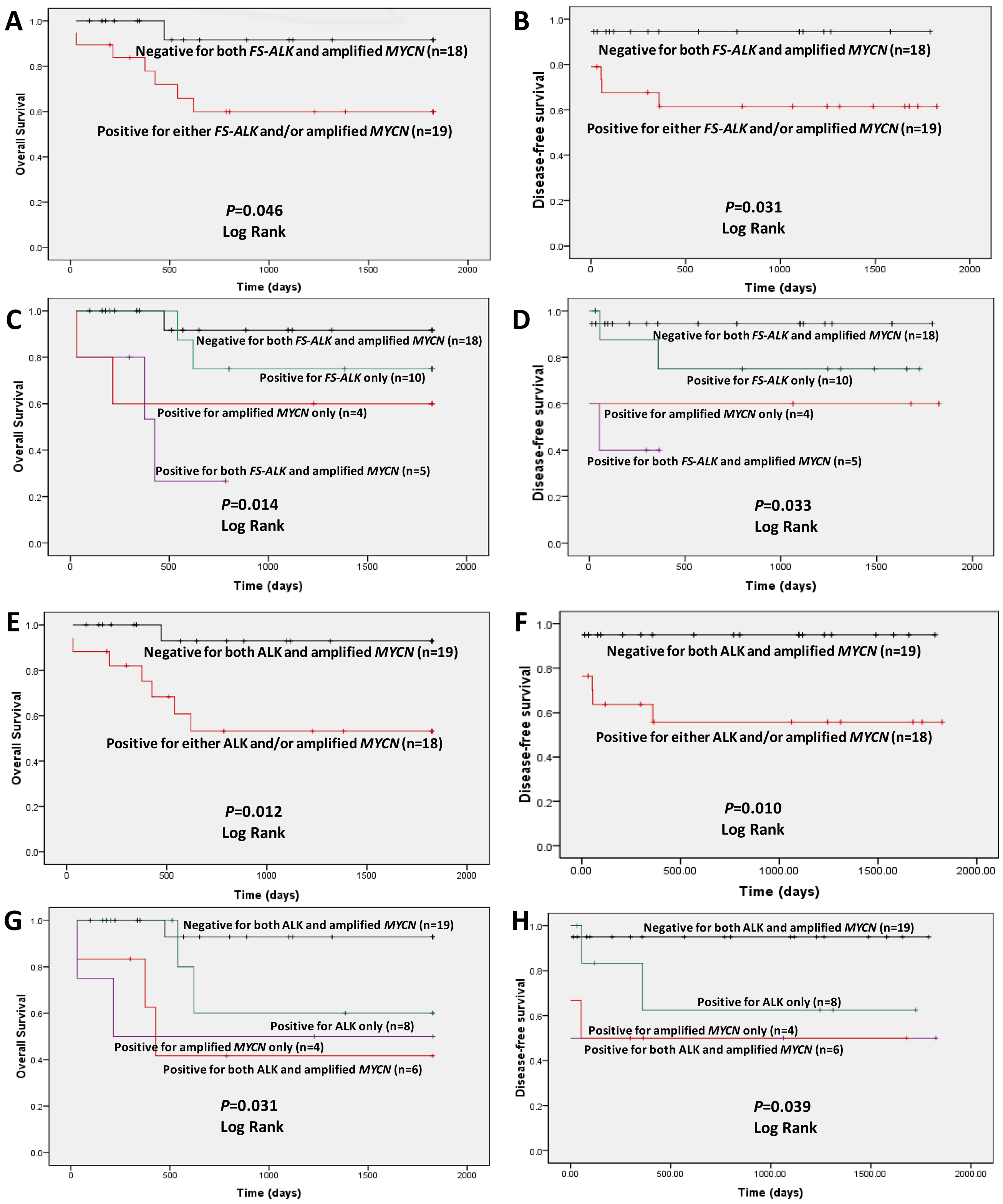

Supplementary Figure 4: *FS-ALK* detectable by RT-PCR and ALK expression detectable by immunohistochemistry are prognostically significant in NB patients based on the overall survival (left panel) and the disease free survival (right panel). Kaplan–Meier survival is illustrated for the following: (**A** and **B**) patients with *FS-ALK* and/or *MYCN* amplification versus those negative for both markers; (**C** and **D**) patients with *FS-ALK* versus patients with *MYCN* amplification versus patients with both markers versus those negative for both markers. (**E** and **F**) patients with ALK and/or *MYCN* amplification versus those negative for both markers; (**G** and **H**) patients with ALK versus patients with *MYCN* amplification versus patients with both markers versus those negative for both markers.
